# Comparing Risk Profiles in Critical Care Patients With Stage 2 and Deep Tissue Pressure Injuries: Exploratory Retrospective Cohort Study

**DOI:** 10.2196/29757

**Published:** 2021-08-27

**Authors:** Jenny Alderden, Linda Amoafo, Yue Zhang, Caroline Fife, David Yap, Tracey Yap

**Affiliations:** 1 University of Utah Salt Lake City, UT United States; 2 Intellicure, Inc The Woodlands, TX United States; 3 Duke University Durham, NC United States

**Keywords:** pressure ulcer, pressure injury, critical care, intensive care, hospital-acquired pressure injury, tissue damage, electronic health records, EHRs

## Abstract

**Background:**

Understanding hospital-acquired pressure injury (HAPrI) etiology is essential for developing effective preventive interventions. Pressure injuries are classified based on the degree of visible tissue damage; the two most commonly identified HAPrI stages in critical care patients are stage 2 and deep tissue injury (DTI). Some experts speculate that stage 2 and DTI have different etiologies, with stage 2 injuries formed from the “outside in” as a result of tissue deformation, decreased perfusion, and subsequent ischemia caused by external pressure and/or shear forces, whereas DTI emerges from the “inside out” due to inadequate perfusion to the deeper tissues causing tissue ischemia.

**Objective:**

The purpose of this study was to compare risk profiles of intensive care unit (ICU) patients who developed stage 2 injuries versus DTIs.

**Methods:**

This was a retrospective cohort study to compare the risk profiles of patients in the ICU with stage 2 injuries and DTIs using electronic health record data. Eligible patients were admitted to the surgical or cardiovascular ICU at an academic medical center in the United States between 2014 and 2018. Anatomic locations were examined, and differences in anatomic patterns were compared using the *χ^2^* test. Risk profile variables included demographic characteristics, Braden Scale scores, vasopressor infusions, hypotension, surgical factors, length of stay, BMI, laboratory values, diabetes, Charlson Comorbidity Index, and the levels of sedation or agitation. The distributions of potential risk variables between patients with stage 2 injuries and DTIs were summarized and compared. A logistic regression model with the least absolute shrinkage and selection operator method was developed to identify the critical risk factors for distinguishing stage 2 and DTI patients.

**Results:**

A total of 244 patients developed a stage 2 injury or DTI during the study period. Of those, 38 patients with medical device–related pressure injury were excluded. The final study sample consisted of 206 patients (n=146 stage 2 and n=60 DTI). Compared with DTIs, stage 2 HAPrIs were more likely to be located on a bony prominence (n=206, *χ*^2^_1_=8.43, *P*=.03). The multivariate model showed that patients who developed stage 2 HAPrIs had a longer length of stay in the ICU than those with DTIs (odds ratio [OR] 1.001, 95% CI 1-1.002, *P*=.03) but were less likely than patients with DTIs to experience a diastolic blood pressure <50 mmHg (OR 0.179, 95% CI 0.072-0.416, *P*<.001) or receive an epinephrine infusion (OR 0.316, 95% CI 0.079-0.525, *P*=.008).

**Conclusions:**

Stage 2 injuries and DTIs have different risk factors and different anatomic patterns. Patients who developed DTIs were more likely to experience low diastolic blood pressure and receive epinephrine, a potent vasopressor. Stage 2 injuries were more likely to occur on the bony prominences, whereas DTIs commonly occurred on the fleshy parts of the body such as the buttock.

## Introduction

The United States has an estimated cost burden exceeding US $26 billion for hospital-acquired pressure injury (HAPrI) treatment [1], although these injuries are considered to be mainly preventable. The prevailing belief about the development of HAPrIs is that a localized injury occurs to an area of the skin and underlying tissue—usually over a bony prominence—as a result of external pressure and sometimes shear forces, along with additional factors that have yet to be elucidated [2]. Patients admitted to the intensive care unit (ICU) are more likely than other hospitalized patients to experience hemodynamic instability and hypotension; in fact, their risk tends to double [3] given a constellation of other factors such as longer length of stay [4,5], surgical factors [6], vasopressor infusions [7,8] older age [9], increased severity of illness [10], and decreased mobility [8,9].

Understanding the etiology of HAPrI is foundational for developing effective preventive interventions. The National Pressure Injury Advisory Panel and the European Pressure Ulcer Advisory Panel provide a pressure injury (PrI) classification staging system (stages 1-4, deep tissue injury [DTI], and unstageable) along with common descriptions of the extent of the visible skin and tissue damage for the purpose of clinical practice, audits, and research [2]. Stage 2 and DTI, the two most commonly occurring injuries within the classification system, appear to be markedly different: stage 2 injuries are generally shallow ulcers, whereas DTIs present as discolored intact skin as a result of damage to the underlying tissue. Some experts speculate that stage 2 injuries and those considered to be DTIs have different etiologies, with stage 2 injuries forming from the “outside in” as a result of tissue deformation, decreased perfusion, and subsequent ischemia caused by external pressure and/or shear forces, whereas DTIs emerge from the “inside out” due to inadequate perfusion to the deeper tissues causing tissue ischemia [11]. If a stage 2 injury occurs from the “outside in” and a DTI forms from the “inside out,” it is likely that ICU patients with a DTI would experience more perfusion-related risk factors. Therefore, the purpose of this study was to compare the risk profiles of ICU patients who developed stage 2 injuries and DTIs.

## Methods

### Design

This was a retrospective cohort study of electronic health record (EHR) data to establish risk factor profiles of ICU patients comparing stage 2 injuries and DTIs. The institutional review board of the University of Utah approved the study (IRB_00111380).

### Sample and Setting

Eligible patients were those who were admitted to the surgical or cardiovascular ICU at an academic medical center in the United States between 2014 and 2018. Inclusion criteria were age >18 years and development of a nonmedical device–related stage 2 injury or DTI during the ICU stay. Exclusion criteria were pediatric patients (<18 years old) and the absence of an ICU-acquired stage 2 injury or DTI. Patients with a medical device–related PrI were included in the study only if they also developed a nonmedical device–related PrI of stage 2 or a DTI [12]. Patients with PrI present on admission to the ICU were only included in the study if they also subsequently developed an ICU-acquired PrI.

Critical care nurses at this institution conduct a head-to-toe skin assessment each shift and document results in the EHR, noting the location and stage of any HAPrI, which is then confirmed by a certified wound care nurse. Patients unable to reposition independently are repositioned every 2 hours, and skincare protocols are employed to encourage nurses to keep the skin as clean and dry as possible. All of the ICU beds are pressure redistribution/low air loss beds; bariatric pressure redistribution/low air loss beds are used for patients with obesity.

### Data Collection

Data were obtained using a combination of a query of the institution’s critical-care datamart cross-referenced with data maintained by the institution’s quality department, along with a manual review of the EPIC EHR system when necessary. Data extracted were limited to the first ICU stay for patients with multiple ICU stays. To capture HAPrI risk factors, only data from the timeframe between ICU admission and HAPrI detection were included.

### Measures

Stage 2 injury and DTI were defined according to the National Pressure Injury Advisory Panel definitions [13]; a PrI was considered to be hospital-acquired if it was detected ≥48 hours after admission. The anatomic location was recorded from structured fields in the EHR and also verified with photographic evidence when available. Risk profile variables included demographic characteristics [14], Braden Scale scores [15], vasopressor infusions [7], hypotension [16], surgical factors [6,16,17], length of stay [5,18], BMI [19], laboratory values [16], diabetes [4], Charlson Comorbidity Index (categorizing comorbidities of the patients’ disease burden and their 1-year mortality risk) [20], and the Riker score (levels of sedation or agitation) [21].

### Analysis

All statistical analyses were performed in R version 3.6.1. (R Foundation for Statistical Computing). First, the distributions of potential risk variables between patients with stage 2 injuries and DTIs were summarized and compared using *t* tests (or Wilcoxon rank-sum tests) for continuous variables and with the *χ^2^* test (or Fisher exact test) for categorical variables. Variance inflation factors (VIFs) were calculated to detect if multicollinearity was present among the list of potential predictors. If none of the potential predictors had a VIF exceeding 5, we treated all predictors as independent predictors. We employed a logistic regression model with the least absolute shrinkage and selection operator (LASSO) method [22] to identify the critical risk factors for distinguishing stage 2 injury and DTI patients.

Unlike other variable selection approaches—such as the stepwise approach—the LASSO approach does not select important risk factors based on *P* values. Instead, by imposing a penalty in the regression model fitting, the LASSO approach shrinks the coefficients of unimportant predictors to zero while retaining the important coefficients [22]. The optimal penalty term was determined using 10-fold cross-validation criteria; the predictor was selected if, and only if, its coefficient was nonzero. The final model included all important predictors with parsimonious representation, enhanced interpretability, and improved precision. From the soft-thresholding property of LASSO in generalized linear regression models, the estimated regression coefficients from the penalized logistic regression were biased toward zero. To mitigate these bias problems, we report a more unbiased estimation of the regression coefficients from unpenalized logistic regression using the selected factors from the LASSO.

## Results

A total of 244 patients developed a stage 2 injury or DTI during the study period. Of those, 38 patients with a medical device–related PrI were excluded. The final study sample consisted of 206 patients (n=146 stage 2 and n=60 DTI). Univariate relationships between study variables and HAPrI stage are presented in Table 1. The multivariate model for the effects of risk profiles on stage 2 versus DTI development is presented in Table 2.

Compared with DTIs, stage 2 HAPrIs were more likely to be located on a bony prominence (n=206, *χ^2^*_1_=8.43, *P*=.03). Among the 146 stage 2 HAPrIs, 93 (63.7%) were located on the sacrum or the coccyx and 11 (7.5%) were located on other bony prominences (ischium, heel, or spine). Among the 60 DTIs, 30 (50%) were found on a bony prominence (sacrum, coccyx, ischium, heel, or spine), whereas the other 30 (50%) occurred on fleshy parts of the body, particularly the buttock (n=23, 38%).

**Table 1:** Risk factor profiles in stage 2 and deep tissue hospital-acquired pressure injury.

Variable		Stage 2 injury (n=146)	Deep tissue injury (n=60)	*P* value
**Demographic factors**				
	Age (years), mean (SD)	57.5 (16.4)	57.5 (14.0)	.99^a^
	Sex (male), n (%)	86 (58.9)	43 (71.7)	.09^b^
	Race (White), n (%)	117 (80.1)	48 (80.0)	.98^b^
	Ethnicity (Non-Hispanic), n (%)	123 (84.2)	50 (83.3)	.87^b^
	Hospital length of stay (days), mean (SD)	31 (23)	28 (19)	.52^c^
	ICU^d^ length of stay (days), mean (SD)	20 (18)	18 (18)	.35^c^
**Surgical factors, mean (SD)**				
	Longest surgery (hours)	3 (3)	5 (4)	<.001^c^
	Total surgical time (hours)	6 (4)	4 (5)	.001^c^
**Laboratory values, mean (SD)**				
	Minimum albumin (g/dl)	2.7 (0.7)	2.6 (0.5)	.23^a^
	Minimum hemoglobin (g/dl)	7.5 (2.1)	7.4 (2.2)	.76^a^
	Maximum lactate (mmol/l)	7.3 (6.0)	5.1 (4.1)	.008^c^
	Maximum creatinine mg/dL	2.9 (2.1)	2.9 (2.2)	.78^c^
**Blood pressure, n (%)**				
	Systolic <90 mmHg	120 (82.2)	54 (90.0)	.16^b^
	MAP^e^ <60 mmHg	99 (67.8)	43 (71.7)	.59^b^
	Diastolic <50 mmHg	60 (41.1)	44 (73.3)	<.001^b^
	Epinephrine	49 (33.6)	40 (66.7)	<.001^b^
	Norepinephrine	71 (48.6)	33 (55.0)	.41^b^
	Dopamine	12 (8.2)	2 (3.3)	.36^f^
	Vasopressin	63 (43.2)	41 (68.3)	.001^b^
	Phenylephrine	8 (5.5)	3 (5.0)	.99^b^
**Diagnosis and comorbidities**				
	Charlson Comorbidity Index, mean (SD)	4.3 (2.9)	4.5 (2.7)	.53^c^
	Diabetes, n (%)	28 (19.2)	14 (23.3)	.50^b^
Minimum Braden scale score, mean (SD)		11.1 (3.0)	11.5 (2.8)	.32^c^
Minimum Riker score, mean (SD)		2.2 (1.2)	1.8 (1.0)	.08^c^

^a^Based on the *t* test.

^b^Based on the *χ^2^* test.

^c^Based on the Wilcoxon rank-sum test.

^d^ICU: intensive care unit.

^e^MAP: mean arterial pressure.

^f^Based on the Fisher exact test.

**Table 2:** Logistic regression model for stage 2 vs deep tissue injury after least absolute shrinkage and selection operator variable selection.

Variable	Odds ratio	95% CI	*P* value
Sex (reference: male)	0.335	0.14-0.755	.11
Hospital length of stay (days)	1.001	1-1.002	.03
Longest surgery (hours)	0.998	0.996-1	.06
Minimum albumin (g/dl)	0.696	0.353-1.351	.29
Diastolic blood pressure <50 mmHg	0.179	0.072-0.416	<.001
Epinephrine	0.316	0.079-0.525	.008
Dopamine	4.277	0.836-33.803	.11

## Discussion

### Principal Findings

This exploratory study investigated differences in risk factors and anatomic patterns between surgical and cardiovascular ICU patients who developed stage 2 injuries vs DTIs. Understanding HAPrI risk factors and the associated etiology is essential for developing effective prevention approaches. It is possible that etiological differences exist between different PrI stages, with stage 2 PrI resulting primarily from external pressure and/or shearing forces affecting perfusion to the superficial tissues and DTI resulting from inadequate blood flow (perfusion) to the deeper tissues [23]. ICU patients are an ideal population for studying perfusion in relation to HAPrIs because they are more likely to experience hypotension and hemodynamic instability.

In this exploratory study, low diastolic blood pressure had the strongest correlation with the development of a DTI. This finding is consistent with a prior study aimed at identifying risk factors for DTI in ICU patients [16], which found that for every 1 mmHg decrease in diastolic blood pressure, the odds of a DTI increased by about 8%. Considering that the heart is in diastole about 2/3 of the time (and in systole 1/3 of the time), it is logical that inadequate diastolic blood pressure is a significant factor contributing to tissue perfusion.

The finding that epinephrine, a potent vasopressor, was associated with DTI is further evidence for the importance of tissue perfusion in DTI etiology. Vasopressor drugs are administered to improve organ perfusion in patients with hypotension; however, the alpha-adrenergic properties of certain vasopressors, including epinephrine, cause arterial vasoconstriction and therefore decrease blood flow to the vessels in the muscles and peripheral tissues. Although it is well established that vasopressor drugs are risk factors for HAPrI, likely as a result of their indication (severe illness and hypotension) and the mechanism of action (arterial vasoconstriction) [8,9,24], this is the first study to examine vasopressors in relation to HAPrI stage.

This study showed that stage 2 HAPrIs were more likely than DTIs to be located on a bony prominence, whereas DTIs were mostly located on the fleshy parts of the body (primarily the buttock). This finding has important clinical implications for routine nursing care because routine skin assessment usually involves checking the bony prominences and not the fleshy areas [2]. Therefore, the fleshy areas, particularly the buttock, should be included in routine nursing skin assessments.

The differences in anatomic patterns, with stage 2 injuries mostly occurring on the bony prominences and DTIs mainly occurring on the fleshy areas, suggest a potential etiological difference between stage 2 injuries and DTIs. Stage 2 HAPrIs are likely primarily caused by pressure (tissue compression) between a surface (eg, a bed) and a bony prominence, or pressure combined with shear force [2]. However, fleshy areas are typically not exposed to significant external pressure, and therefore an “inside-out” etiology driven by perfusion should be considered [11,16,24].

### Limitations

The study is limited by its small sample size, and therefore the results are considered exploratory. We were limited to the data contained in the EHR, and consequently unable to accurately obtain variables difficult to capture retrospectively, such as repositioning frequency. Moreover, these data are based on a single study site and therefore may not be generalizable to other institutions.

### Conclusion

Results from this exploratory study performed in surgical and cardiovascular ICU patients showed that deep tissue and stage 2 HAPrIs have different risk factors and different anatomic patterns. Patients who developed DTIs were more likely than patients with stage 2 HAPrIs to experience low diastolic blood pressure and to have received epinephrine, a potent vasopressor. Stage 2 HAPrIs were more likely to occur on the bony prominences, whereas DTIs commonly occurred on the fleshy parts of the body such as the buttock. Future research is needed to elucidate the detailed etiologic differences between stages, which will lead to identifying effective preventive interventions.

## Abbreviations

DTI: deep tissue injury
EHR: electronic health record
HAPrI: hospital-acquired pressure injury
ICU: intensive care unit
LASSO: least absolute shrinkage and selection operator
PrI: pressure injury
VIF: variance inflation factor
